# Multiple Mycotoxins in Rice: Occurrence and Health Risk Assessment in Children and Adults of Punjab, Pakistan

**DOI:** 10.3390/toxins10020077

**Published:** 2018-02-10

**Authors:** Saima Majeed, Marthe De Boevre, Sarah De Saeger, Waqar Rauf, Abdul Tawab, Fazal-e-Habib, Moazur Rahman, Mazhar Iqbal

**Affiliations:** 1 Health Biotechnology Division, National Institute for Biotechnology and Genetic Engineering (NIBGE), Faisalabad 38000, Pakistan; saima.nibge@gmail.com (S.M.); wadhamite@gmail.com (W.R.); atawab1@yahoo.com (A.T.); fazal_ehabib@yahoo.com (F.-e.-H.); moazur@yahoo.com (M.R.); 2 Department of Biotechnology NIBGE, Pakistan Institute of Engineering and Applied Sciences, Nilore, 45650, Islamabad, Pakistan; 3 Laboratory of Food Analysis, Department of Bioanalysis, Faculty of Pharmaceutical Sciences, Ghent University, Ghent 9000, Belgium; Marthe.DeBoevre@ugent.be (M.D.B.); sarah.desaeger@ugent.be (S.D.S.)

**Keywords:** LC–MS/MS, consumption, cooking effect, dietary exposure, aflatoxins, margin of exposure, cancer risk

## Abstract

Mycotoxin contamination in rice can create a health risk for the consumers. In this study, the measurement of 23 mycotoxins in rice samples (*n* = 180) was performed using a validated LC–MS/MS method. A food frequency questionnaire was used to get rice consumption data for the assessment of mycotoxin dietary exposure, before calculating the health risk in adults and children of north and south regions of the Pakistani Punjab province. The prevalence of aflatoxin B_1_ (56%), aflatoxin B_2_ (48%), nivalenol (28%), diacetoxyscirpenol (23%), fumonisin B_1_ (42%), zearalenone (15%), HT-2 toxin (10%), deoxynivalenol (8%), and ochratoxin A (6%) was estimated in samples with a mean concentration range between 0.61 and 22.98 µg/kg. Aflatoxin degradation by traditional Pakistani cooking recipes was evaluated and observed to be 41–63%. The dietary exposure to aflatoxins exceeded the tolerable daily intake at all levels, and ochratoxin A and zearalenone posed health risk at high contamination and high consumption levels. The margin of aflatoxin B_1_ exposure ranged between 10 and 69 in adults and 10 and 62 in children. The mean cancer risk by aflatoxin B_1_ exposure was 0.070 (adults) and 0.071 (children) cases/year/100,000 people in South Punjab population, and 0.122 (adults) and 0.127 (children) cases/year/100,000 people in North Punjab population. This study will provide new insights for the planning and management of mycotoxins in Pakistan.

## 1. Introduction

Mycotoxins are naturally occurring toxic secondary metabolites produced by fungal species of the genera *Aspergillus*, *Alternaria*, *Penicillium*, *Fusarium*, *Claviceps*, and several others. Mycotoxins can be nephrotoxic, immunosuppressive, carcinogenic, and teratogenic. Trichothecenes, aflatoxins (AFs), *Alternaria* toxins, fumonisins (FBs), ochratoxin A (OTA), and zearalenone (ZEN) are the most important classes of mycotoxins causing a great variety of toxic effects in humans as well as in animals. Moreover, significant economic losses (25%) occur in global agricultural commodities because of mycotoxin contamination [1].

Rice (*Oryza sativa* L.) is the most important source of human calorie intake and is a staple food in many countries [2]. Rice is one of the main crops of Pakistan with an annual production of 6800 metric ton dehulled rice and exports of about 60% of its annual production [3]. Rice, cultivated in flooded irrigation conditions and high moisture levels, is susceptible to get infected by mold and to subsequent mycotoxin contamination. Also inappropriate storage and climatic conditions such as floods and heavy rainfall at the time of harvest aggravate the situation. Sun-drying of rice, usually practiced by most of the farmers, is insufficient to reduce the moisture content, thus making rice more prone to fungal attack [4,5]. 

The toxic effects caused by mycotoxins in animals and humans are called mycotoxicosis. The severity depends on many factors, especially the toxicity, mycotoxin contamination level, age and health status of the individual, along with possible synergistic effects of other chemicals encountered by the person [6]. The toxic effects range from acute (e.g., liver or kidney deterioration) to chronic (e.g., liver cancer); also mutagenic and teratogenic effects cause symptoms that range from skin irritation to immunosuppression, birth defects, neurotoxicity, and death [7]. More than 80% of hepatocellular carcinoma occur in low-income countries, having high risk factors of dietary aflatoxin exposure and chronic hepatitis B and hepatitis C (HBV and HCV) viral infection [8]. According to the International Agency for Research on Cancer (IARC), aflatoxins are classified in Group 1 (carcinogenic to humans), whereas fumonisin B_1_ (FB_1_) is classified in Group 2B (possibly carcinogenic to humans). Deoxynivalenol (DON) and other trichothecenes, as well as AFB_1_ can exert immunosuppressive effects, and FB_1_ may contribute to neural tube defects. Renal dysfunction due to OTA exposure is also a significant problem, especially because of the potential of the toxin to exacerbate impaired renal function in individuals with diabetes [9,10]. 

To reduce the risk of mycotoxin consumption, legislative measures in different countries (e.g., EU, Japan, Korea, and China) have been setting the maximum permissible limits (ML) for different mycotoxins in food and feed. The European Commission has established the regulatory limits for mycotoxins in ready-to-use rice as follows: aflatoxin B_1_ (2 µg/kg), total AFs (4 µg/kg), OTA (3 µg/kg), DON (750 µg/kg), ZEN (75 µg/kg), and an indicative value (50 µg/kg) for the sum of T-2 toxin (T2) and HT-2 toxin (HT2), while, for unprocessed rice, the established maximum limits are of 5 µg/kg for AFB_1_, 10 µg/kg for the sum of aflatoxins (AFs), 5 µg/kg for OTA, 1250 µg/kg for DON, 100 µg/kg for ZEN, and 100 µg/kg for ^the^ sum of T2 and HT2 toxin [11,12]. 

Risk assessment is the combination of hazard identification and its characterization, exposure assessments, and subsequent risk characterization [13]. Many mycotoxins and toxigenic fungi have been identified as hazardous for health [14]. The characterization of hazards involve the measurement of the adverse health effects (both chronic and acute effects) caused by mycotoxins. For noncarcinogenic mycotoxins, a tolerable daily intake (TDI) or health-based guidance value (HBGV) has been established [15]. There is no acceptable daily intake (ADI) for aflatoxins as they are genotoxic and carcinogenic. If daily consumption is below the TDI values of mycotoxins, which is 1000 ng/kg body weight (b.w.) day for DON [16], 1200 ng/kg b.w. day for NIV [17], 100 ng/kg b.w. day for the sum of T-2 and HT-2 [18], 250 ng/kg b.w. day for ZEN [19], 2000 ng/kg b.w. day for FBs [20], and 17 ng/kg b.w. day for OTA [21], no adverse human health effects would appear over a life time. 

Mycotoxin exposure assessment depends on the concentration of mycotoxins in food and on its intake. Risk characterization of nongenotoxic mycotoxins is done by comparing their exposure assessment with TDI. The risk characterization of genotoxic aflatoxins is usually executed by two approaches. The first one calculates cancer risk considering the prevalence of Hepatitis B Virus (HBV), on the basis of Hepatitis B surface antigen-postitive individuals (HBsAg^+^ individuals) in a given population and the cancer potency of aflatoxins in HBV carriers (0.3 cancers/year per 100,000 people per ng aflatoxin/kg b.w. day) and noncarriers (0.01 cancers/year per 100,000 people per ng aflatoxin/kg b.w. day) [22]. The second approach calculates the margin of exposure (MoE), which is the ratio between the toxicological threshold derived from animal studies and the estimated exposure in humans [23]. 

In Pakistan, the previous studies are mostly restricted to the determination of aflatoxins, zearalenone, and ochratoxin A contamination using nonconfirmatory analytical techniques [24,25,26,27,28,29,30,31], while mycotoxin contamination in rice at a global level has been reviewed by Ferre et al. and Tanaka et al. [32,33]. The studies on the dietary exposure to mycotoxins through rice consumption were performed in different countries, like Iran [34], United States [35], Turkey [36], Nigeria [37], France [38,39], Thailand [40], Korea [41], Vietnam [42,43], Mediterranean region [44], Africa [45], Spain [46], West Africa [47], and Portugal [48].

Limited data is available on the determination of and dietary exposure to multi-mycotoxins in Pakistani rice. Previous studies embarked upon the occurrence and dietary exposure to only a few mycotoxins (AFs, OTA, and ZEN) [25,26,30,31]. These studies did not include the dietary exposures to multiple mycotoxins, especially in children, are devoid of important parameters such as the calculation of the margin of exposure (MoE), and did not include a food frequency questionnaire (FFQ) and the degradation effect of Pakistani cooking recipes on the prevalent mycotoxins. 

To the best of our knowledge, no study has been published on the simultaneous determination of multi-mycotoxins covering aflatoxins (AFB_1_, AFB_2_, AFG_1_, AFG_2_), OTA, fumonisins (FB_1_ and FB_2_), DON, nivalenol (NIV), diacetoxyscirpenol (DAS), HT2, and ZEN in Pakistani rice samples as well as on their dietary exposure in adults and children of Pakistan using confirmatory analytical techniques. Hence, to fill the aforementioned gaps, this work aimed to study the contamination levels of multi-mycotoxins in rice and their exposure risk assessment, based on rice consumption data in different regions. After accounting for the degradation effect of Pakistani cooking recipes on the prevalent mycotoxins, the study subsequently enabled us to determine the dietary exposure to mycotoxins in two population groups (adults and children), as the intake exceeding the provisional maximum tolerable daily intakes (PMTDIs) set by the European Food Safety Authority (EFSA) that poses health risks. Also, the potential risk of liver cancer arising from aflatoxin B_1_ intake was assessed in the Pakistani population of South Punjab (SP) and North Punjab (NP) regions. The study is likely to contribute to devising strategies for the planning and management of these food contaminants for the Pakistani Punjab population.

## 2. Results

### 2.1. Validation 

The validation parameters were calculated from the relative peak area, using deepoxy-deoxynivalenol (DOM) as an internal standard for NIV, DON, fusarenon-X (FUS-X), neosolaniol (NEO), 3-acetyl-deoxynivalenol (3-ADON), 15-acetyl-deoxynivalenol (15-ADON), HT2, T2, and DAS, while zearalanone (ZAN) was used for the rest of the analytes. The validation parameters were calculated in terms of linearity, limit of detection (LOD), limit of quantification (LOQ) (Table 1), % apparent recovery, intraday repeatability, interday repeatability, and expanded measurement uncertainty (Table A1). 

There were no interfering peaks at the retention times of all the analytes. The specificity was checked by running 20 blank rice samples on LC–MS/MS. The blank rice samples were spiked at five calibration levels with each compound, and the calibration curves were prepared each day in triplicate for four validation days. For the samples in which the contamination level did not fall in this validated range, either lower or higher range calibration curves were plotted to quantify them. The method-matched calibration curves fitted by linear regression showed a coefficient of determination (R^2^) ranging from 0.991 to 0.999. The mean recoveries for all the analytes were in the range of 80% to 111% (Table A1). The relative standard deviation was calculated in intraday repeatability (RSD_r_) and interday repeatability (RSD_R_) conditions and ranged from 3% to 14% and from 6% to 20%, respectively. The LOD values of all the mycotoxins ranged from 0.5 to 53 µg/kg. The LOQs for all the toxins ranged from 1.5 to 105 µg/kg. The expanded measurement uncertainty ranged from 2.01% to 34.4%. Method-matched calibration curves were used for the calculation of LOD and LOQ. The ion ratios of the qualifier and the quantifier ions were also monitored to successfully qualify the EU criteria to confirm the positive identification of a compound. The validation results of the current multi-mycotoxin method matched with the required European Commission Performance Criteria [49].

### 2.2. Mycotoxin Levels 

The validated method was further used to investigate the incidence and content of mycotoxins in the collected rice samples. The results of the occurrence and levels of mycotoxins in rice samples are summarized in Table 2. Here, the mean is the average of mycotoxin contamination levels in all the samples including the negative samples, while considering their level as zero. The frequency of contamination of samples with AFB_2_ and AFB_1_ was 48% and 56%, respectively, within the concentration range of 1.5–9.15 µg/kg and 1.5–40 µg/kg, respectively. AFG_1_ and AFG_2_ were not detected in the tested samples. The results indicated that FB_1_ was found in 42% (level range from 26 to 75 µg/kg) and ZEN in 15% (level range from 13 to 114 µg/kg) of the collected samples. OTA was detected in 6% of the samples within a concentration range of 2.5–11 µg/kg. Trichothecenes including DON, NIV, HT2, and DAS occurred in 8%, 28%, 10%, and 23% samples, and the mean contamination level of these mycotoxins were 6.99, 13.9, 2.03, and 1.60 µg/kg, respectively. 

**Figure 1:**
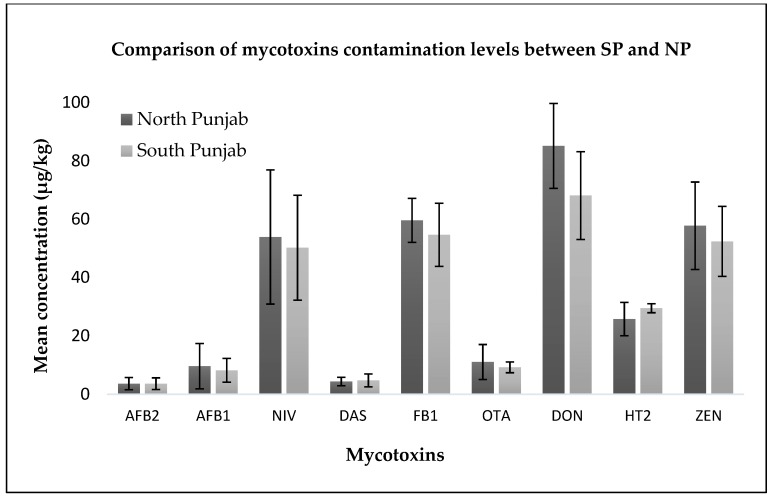
Comparison of the mean contamination levels of different mycotoxins in positive samples of the examined regions (NP: North Punjab; SP: South Punjab).

Figure 1 shows the mean contamination levels in the positive samples only (above the LOQ). The incidence rate of the mycotoxins was higher in North Punjab than in South Punjab. The levels of contamination of mycotoxins in both regions were not significantly different (*p* < 0.05). Overall, 78% of the samples were contaminated with either one or more mycotoxins, while 57% of the samples were contaminated with more than one mycotoxins. In 40% of the samples, AFB_1_ and AFB_2_ co-occurred. In 21% of the samples, the co-occurrence of the mycotoxins produced from *Fusarium* and *Aspergillus* moulds was observed. The co-occurrence of these specific toxins can be taken into account in the design of future toxicity studies to examine their impact on animal and human health. 

### 2.3. Consumption of Rice 

On the basis of this survey, the mean rice consumption in the adult population (16–65 years) at national level was 108 ± 24.1 g per individual on a daily basis, while the rice intake (g) per kg of body weight (b.w.) was 1.92 ± 0.624 g (Table 3). Similarly, the mean rice consumption per capita and daily intake of rice per kg of body weight in children (7–15 years) at national level were estimated to be 56.65 ± 15.3 g/individual/day and 1.97 ± 0.619 g/kg b.w./day, respectively. The difference in rice consumption between adults and children of both regions was not significant (*p* < 0.05). In all the cases, the mean at national level was also not significantly different.

### 2.4. Effect of Cooking on AFB_1_ and AFB_2_ Levels 

During cooking, the first step involved washing that caused a 15% reduction of aflatoxins. The reduction in AFB_1_ (41–51%) and AFB_2_ (47–63%) in three cooking recipes of rice was measured (Table 4). Aflatoxin reduction was significantly higher (*p* < 0.05) in Biryani than in other cooking recipes. AFB_1_ reduction after cooking Pulao was in parallel with the reduction in boiled rice. The reduction level was different among these three cooking recipes because of differences in the cooking method. In boiled rice, the excess water was kept without adding any spices. In contrast, in Biryani recipe, the excess water was drained out after boiling the rice, spices (Coriander seed, cloves, cinnamon, ginger, black pepper, cardamom black, dry curry leaves, chili, and cumin seed) were added, and the rice was further cooked. 

### 2.5. Exposure Assessment of Mycotoxins in Pakistani Children and Adults 

The deterministic exposure levels associated with the mycotoxins AFB_2_, AFB_1_, NIV, DAS, FB_1_, OTA, DON, HT2 toxin, and ZEN are shown in Table 5 and Table 6. The dietary exposure levels of AFB_1_ and AFB_2_ were taken after considering the mean reduction by cooking. We considered the two approaches, the mean concentration approach using fixed mycotoxin concentrations and variable values (mean, median, maximum, and percentiles) of consumption, and the second approach using a fixed consumption level with variable values (mean, median, maximum, and percentile) of mycotoxin level. In each approach, three scenarios, i.e., upper bound (<LOQ = LOQ), medium bound (<LOQ = ½ LOQ), and lower bound (<LOQ = 0) were calculated, and the results of the medium bound are shown in Table 5 and Table 6, while the results of the other two scenarios are given as supplementary material (Table A2, Table A3, Table A4 and Table A5).

In this study, the estimated AFB_1_ and AFB_2_ intake in Pakistani adults and children through rice consumption was above the safe limits at all calculated exposure levels. The exposure of OTA for both adults and children of SP and of NP exceeded the PMTDI of 17.1 ng/kg b.w./day only at the maximum level [21]. There was no health risk associated with NIV exposure at the measured contamination levels as none of the exposure levels exceeded the safe health limits for NIV (1200 ng/kg b.w./day) [17]. 

All the determined exposure levels of HT2-toxin were found lower than the PMTDI value of 100 ng/kg b.w./day [18]. The exposure levels of DAS were compared with the TDI values of HT2 and T2 because of their structural similarity. All the estimated exposure levels of DAS, including the maximum exposure, were lower than the PMTDI value of HT2 and T2 (100 ng/kg b.w./day) [17]. However, the consumers’ maximum exposure level for ZEN in NP showed a risk, as the values were higher than the TDI value (250 ng/kg b.w. day) [19]. 

Finally, when the two groups of subjects (adults and children) were compared, the children were found to be a vulnerable group for most contaminants, as the exposure was expressed in terms of per kg of body weight. The study reports, for the first time, the multiple-mycotoxins prevalence in Pakistani rice in combination with a consumption pattern of rice throughout Punjab, Pakistan, and the degradation of aflatoxins by the local cooking methods. This study also provides the first insight on the exposure to multiple mycotoxins associated with rice intake in Pakistan. The exposure of rice consumers in Pakistan (adults and children) to aflatoxins is above the toxicological reference values of AFs at all consumption levels. A combined intake of different mycotoxins at different concentration levels may lead to a higher risk than their single intake [50]. Nonetheless, we can conclude that the dietary exposures associated with the consumption of rice are considered a risk for public health. Hence, management strategies need to focus more on the reduction of mycotoxin contamination.

### 2.6. Cancer Risk and Margin of Exposure

The risk characterization due to AFB_1_ exposure by rice consumption was measured using the margin of exposure (Table 7) and the liver cancer risk approach (Table 8). The margin of exposure was calculated using Bench Mark Dose Level for 10% increased cancer risk based on the rodent data (BMDL_10_) which is 170 ng/kg b.w./day [51]. An MoE greater than 10,000 is considered a low health concern [23]. The MoE values were calculated at exposures of fixed AFB_1_ concentration and variable rice consumption by adults and children of SP and NP at lower bound (LB) and upper bound (UB) scenarios (Table 7). According to the results, the population in NP is at a higher risk compared to that of SP. In addition, all the MoE values were <10,000 in both regions, indicating a high risk due to AFB_1_ exposure through rice consumption in Pakistan. 

AFB_1_, a potent human carcinogen, is synergistically linked to human primary liver cancer, highlighting a cancer burden in developing countries. The risk of liver cancer in individuals exposed to chronic hepatitis B virus (HBV) infection together with aflatoxins is 30 times higher than the risk in individuals exposed to aflatoxins only [52]. The age standardized rate (ASR) of liver cancer (HCC) is 3.6 cases per 100,000 individuals per year in the Pakistani population, with a 2.9% incidence [53]. The weighted average of hepatitis B antigen prevalence was 2.4% (range 1.7–5.5%) in children and 2.4% (range 1.4–11%) in the adult population [54]. Taking the HBV prevalence rate in Pakistan into account, the risk estimates of aflatoxin-induced HCC cases were calculated by the deterministic approach using a fixed mean AFB_1_ concentration and a variable consumption. 

The calculated mean cancer risk in adult and child populations of SP in Pakistan were 0.070 and 0.071 cases per year per 10^5^ individuals, respectively, whereas the risk of development of liver cancer in adults and children of NP were calculated to be 0.122 and 0.127 cancers/year/10^5^/ng AFB_1_/kg b.w./day, respectively. The results showed that, at maximum, the total liver cancer risk associated with rice consumption in Pakistan was 0.725 and 0.752 HCC cases/year/100,000 individuals in adults and children, respectively.

## 3. Discussion

### 3.1. Mycotoxin Contamination 

The natural occurrence of multi-mycotoxins in rice has been studied in many regions of the world [32,33]. The present work is the first study on a multi-mycotoxin survey in rice in Pakistan. This work investigates the incidence of the major *Aspergillus* and *Fusarium* mycotoxins, as well as of the minor *Alternaria* mycotoxins in rice by using LC–MS/MS. In previous studies on Pakistani rice [24,25,26,27,28,29,30,31], information about only the traditional mycotoxins, such as aflatoxins and ochratoxin A, was reported. The occurrence of AFB_1_ and OTA reported in Pakistani rice was more or less similar [26,29]. A previous study on Pakistani rice (*n* = 62) recorded that 47% and 37% of samples contained AFB_1_ and OTA in a concentration range of 0.04–21.3 and 0.6–25.4 µg/kg, respectively [26]. Furthermore, our earlier study demonstrated that 56 and 50% of rice samples were positive for aflatoxins and OTA, with levels of contamination ranging from 0.05 to 24 and 0.06 to 15 µg/kg, respectively [29]. 

Studies on multi-mycotoxin contamination of rice, however, have been conducted in some other countries [4,55,56,57,58,59]. A high incidence of multiple mycotoxins was found in Nigerian, Turkish, Chinese, and Indian rice [4,56,58]. A study showed 100% frequency of positive samples (*n* = 21) for AFs in Nigerian rice. The levels of AFB_1_ and AFB_2_ were in the range of 4.1–309 µg/kg (37.2 µg/kg mean level) and 1.3–24.2 µg/kg (mean level of 8.3 µg/kg), respectively. OTA level was in the range of <LOD—341.3 µg/kg in 67% of the samples, with a mean level of 141.7 µg/kg, while ZEN was prevalent in 52% of the samples, with a mean level of 10.6 µg/kg (in the range of <LOD—41.9 µg/kg). The contamination level for DON was in the range of <LOD—112.2 µg/kg in 24% of the samples (mean 18.9 µg/kg). Moreover, 14% of the samples were positive for FB_1_, with concentrations in the range of 0.4–4.4 µg/kg and a mean of 0.2 µg/kg [58]. The levels of AFs and OTA contamination in Turkish rice were found to be in the range of 0.05 to 21.4 and 0.025 to 80.7 µg/kg, with a 58% and 72% incidence rate, respectively [56]. The data from an Indian study revealed the content of AFB_1_ to be in the range of 0.1 to 308 µg/kg in 68% of rice samples (*n* = 1600) [4].

Some studies also reported lower contamination levels of mycotoxins in rice in different countries as compared to the present study [55,57]. A study on rice samples (*n* = 9) from Qatar, observed levels of AFs in the range of 0.14–0.24 µg/kg and of OTA in the range of 1.65–1.95 µg/kg. The level of DON found in one sample was 142.31 µg/kg and the level of ZEN was from 0.18 to 1.41 µg/kg [55]. A survey of rice samples (*n* = 199) sold in Canada revealed that 50%, 22%, and 8% of rice samples were contaminated with AFB_1_, OTA, and FB_1_, respectively. The concentration levels of AFB_1_, OTA, and FB_1_ were lower as compared to the present study, ranging between 0.002–7.1 µg/kg, 0.05–0.49 µg/kg, and 0.7–14 µg/kg, respectively [57].

In the present study, a higher incidence of Aspergillus toxins was observed in comparison to Fusarium mycotoxins. However, in Korean brown rice, an opposite trend was reported, as the frequencies of AFs, ZEN, NIV, and DON were 7.5%, 84%, 29%, and 1.3%, respectively, whereas the levels of contamination were estimated from 0.7 to 2.7 µg/kg (AFs), from 4.2 to 201.3 µg/kg (ZEN), from 7.7 to 349 µg/kg (NIV), and 43.2 µg/kg (DON) [59]. The variation in mycotoxin contamination in rice from different regions may be due to differences in climate as well as differences in the prevalent toxigenic microflora influenced by different agricultural practices and storage conditions [33]. 

### 3.2. Consumption of Rice

Consumption of rice in Iran (107 g/day per person) [34], Japan (157 g/day per capita), and China (183 g/day per capita) are comparable to our consumption results but not to the very high rice consumption (377 g/head/day) in Thailand [60].

### 3.3. Effect of Cooking on AFB_1_ and AFB_2_ Levels 

The reduction of aflatoxins during the cooking process might occur because of its removal during washing, binding to a food matrix, or degradation or modification to unknown products during the aqueous heating process. A study reported a 14.7–15.3% reduction of aflatoxins with washing similar to the results of the present study [61]. The reduction of aflatoxins during rice cooking was reported by previous studies [62,63,64,65]. Ordinary cooking of AFB_1_-contaminated rice gave an average reduction of 34% [64], while a 78–88% reduction was observed with pressure cooking [63]. In another study, cooking of AFB_1_-contaminated rice showed 87.5% (cooking in excess water), 84% (ordinary cooking), and 72.5% (microwave cooking) reduction of AFB_1_ [62]. In contrast, a low level of AFB_1_ reduction (24.8%) was observed with two cooking methods common in Iran [65].

### 3.4. Exposure Assessment of Mycotoxins in Pakistani Children and Adults 

Average AFB_1_ intakes of 22.2–22.3 ng/kg b.w./day [26] and 19.1–26.6 ng/kg b.w/ day [25] were reported in previous studies. Our study results showed lower exposure compared to these previous studies, whereas in the high consumption group the exposure values in our study (42.77 and 44.36 ng/kg b.w. day) were higher. This might be due to differences in sampling, levels of contamination of AFs in rice, and consumption data considered during these studies. By comparing the previous studies conducted in different countries, our results match with the estimated mean AFB_1_ exposure due to rice consumption in Korea (0.89–5.37 ng/kg b.w./day) [41], Brazil (6.5–6.6 ng/kg b.w./day) [66], and Nigeria (5.2 ng/kg b.w./day) [37]. In contrast, in Japan, the observed dietary exposure of AFB_1_ was 1.78 and 1.20 ng/kg b.w./day at the 95th percentile level in children aged 7–14 years and in adults, respectively [67]. 

Because of the low contamination levels, the dietary exposures to AFB_1_ from rice and wheat intake in French adults (<LOD—0.018 ng/kg b.w./day) and children (<LOD—0.035 ng/kg b.w./day) [38], as well as in the Lebanese population (0.63–0.66 ng/kg b.w/ day) based on total diet intake were lower than those observe in the present study [68]. The current data is higher than the reported mean dietary exposure to AFB_1_ (0.8 and 0.12 ng/kg b.w./day) by rice consumption in Thailand in the years 2012 and 2013 [40]. Also, the dietary exposure to AFB_1_ (0.033 ng/kg b.w./day) in Morocco [44] and that to total AFs in French adults (0.014 ng/kg b.w./day) and children (0.077 ng/kg b.w./day) [39] are lower than those observed in the current study. On the other hand, the mean AFB_1_ exposure values of 21.7 ng/kg b.w./day in Northern Vietnam adults, 33.7 ng/kg b.w./day in children [43], 22.2 ng/kg b.w./day in adults of Lao Cai province of Vietnam [42], 5.8–76 ng/kg b.w./day (LB-UB) in the Chinese population [69], and 14 ng/kg b.w./day in the African country Gambia were higher than those observed in our study, because of high rice consumption and contamination levels [45].

The mean dietary exposures of 1.50 ng/kg b.w./day of OTA in the Nigerian population [37] and of 4.28 ng/kg b.w./day in the Lebanese population [68] were comparable to thos eobserved in this study. The higher mean dietary exposures to OTA (24.2–24.7 ng/kg b.w./day) by rice consumption reported in Pakistan [26] and in Vietnam (7.9 ng/kg b.w./day) were due to relatively higher contamination levels of OTA in rice [42]. The mean dietary exposure of OTA was higher in Chinese adults (4.62 ng/kg b.w./day) and children (13.9 ng/kg b.w./day) because of a higher consumption level of cereals [70]. The mean OTA exposure values of 0.0004 ng/kg b.w./day in US adults and 0.001 ng/kg b.w./day in US children aged >6–18 years [35], 0.02 ng/kg b.w./day in Turkish rice consumers [36], 0.17 ng/kg b.w./day in Spain [46], 0.08 ng/kg b.w./day in French adults, 0.19 ng/kg b.w./day in French children [40], and 0.0428 ng/kg b.w./day in Moroccans [44] by rice consumption were lower than those reported in our study.

Similar results were found for DON mean dietary exposure of 9.51 ng/kg b.w./day in French adults and 23.7 ng/kg b.w./day in children by rice intake [39], as well as 5.7–6.6 ng/kg b.w./day in French adults and 14.3–16.0 ng/kg b.w./day in children by rice and wheat intake [38]. Through cereal intake, a higher dietary exposure of DON was reported for Chinese adults (309 ng/kg b.w./day) and children (927 ng/kg b.w./day) compared to what found in the present study [70]. The estimated mean exposure values for DON, 5.97 ng/kg b.w./day in the Nigerian population [37] and 0.18 ng/kg b.w./day in the Moroccan population [44], and 1.56 ng/kg b.w./day in the Lebanese population [68] were lower than those reported in the current study data. The mean estimated exposure values of 3.8 ng/kg b.w./day in French adults, 42.5 ng/kg b.w/ day in children [40], 0.042 ng/kg b.w./day in the Moroccan population [45], and 19.13 ng/kg b.w./day in the Nigerian population [37] for FBs by rice intake were lower than those found in the present study. In contrast, a higher FBs mean exposure of 536 ng/kg b.w./day in Northern Vietnam adults and 1019 ng/kg b.w./day in children was reported [43]. 

In the present study, NIV exposure results are similar to those reported for the Nigerian population (20.81 ng/kg b.w./day) [37]. The present study showed a higher level of the estimated mean exposure for NIV by rice intake as compared to that reported in French adults (4.86 ng/kg b.w./day) and children (11.2 ng/kg b.w./day) [39] and in the Moroccan population (6.12 ng/kg b.w./day) [44], as well as to the mean exposure ranges (UB-LB) of NIV in French adults (1.88–2.8 ng/kg b.w./day) and children (4.76–6.43 ng/kg b.w./day) [39]. Data on the exposure to HT-2 and DAS is scant in the literature. In this study, the HT2 toxin dietary intakes were higher than the mean estimated exposure for the sum of HT2 and T2 (0.361 ng/kg b.w./day) in the Moroccan population [44] and the mean exposure ranges (UB-LB) for HT2 in French adults (0.19–1.53 ng/kg b.w./day) and children (0.16–1.42 ng/kg b.w./day) [38]. The mean estimated exposure (0.101 ng/kg b.w./day) of DAS by rice intake in the Moroccan population [44] was lower as compared to our values. 

The mean dietary exposure values of 2 ng/kg b.w./day for ZEN by rice consumption in Iranian people [34] and in French adults (1.63 ng/kg b.w./day) and children (3.75 ng/kg b.w./day) were lower as compared to those found in our study [39]. Also, the mean estimated exposure ranges (UB-LB) for ZEN by rice and wheat products intake in French adults (0.06–0.7 ng/kg b.w./day) and children (0.16–1.42 ng/kg b.w./day) were lower than those observed in the present study [38]. A higher estimated mean exposure of 157.36 ng/kg b.w./day of ZEN was reported in the Nigerian population [37] by rice intake, and of 155 ng/kg b.w./day in Chinese adults and of 464 ng/kg b.w./day in Chinese children through cereal intake [70]. 

These discrepancies in exposure estimations are due to the variability in food consumption levels, differences in contamination data, cooking effects, and differences in the analytical techniques used in the reported studies.

### 3.5. Cancer Risk and Margin of Exposure

Based on these results, it is obvious that the intake of contaminated rice is of great public health concern. Our results for the estimated cancer risk and MoE due to AFB_1_ intake by rice consumption are comparable with those of previous studies reported for Japan [67] and Thailand [40]. The study for Japan measured a cancer risk of 0.031 cancer/year/10^5^, and a MoE of 141 for children (age 7–14 years) resulting from AFB_1_ intake by rice consumption, while in adults the values of cancer risk and margin of exposure were 0.021 cancer/year/10^5^ and 209 MoE, respectively [67]. The mean cancer risk and margin of exposure due to AFB_1_ intake through colored and brown rice in all age groups of Thailand were in the range of 0.010–0.039 cancer/year/10^5^, with 5% HBV prevalence rate [40].

The mean cancer risk and margin of exposure due to total dietary AFB_1_ intake in Brazil [66] and Malaysia [71] were also similar to those determined in our study. The mean cancer risk and margin of exposure in Brazil, with a 0.37% prevalence rate of HBV, were 0.0753 cancer/year/10^5^ and 25 MoE, respectively [66]. The liver cancer risk in Malaysia by total dietary AFB_1_ calculated by eliminating high contamination data was 0.01–0.26 cancer/100,000 people/year, with a 0.2–2.1% contribution to liver cancer cases, while, by adding the high contamination data, the value increased to 0.61–0.85 cancer/year 10^5^ people, raising the contribution to liver cancer cases up to 12.4–17.3% [71]. 

The cancer risk and margin of exposure due to AFB_1_ intake through rice in Chinese [69], Vietnamese [42], and African populations [45] were higher as compared to those found in the present study. This might be because of high contamination levels of AFB_1_ in rice and high consumption levels, as well as high prevalence rate of HBV in those countries. The cancer risk and margin of exposure in the Chinese population were 0.2–2.65 (UB-LB) cancer/year/10^5^ people and 1.8–24.1 (UB-LB) MoE, respectively, using a 14.3% prevalence rate of HBV [69]. The mean cancer risk and margin of exposure in Vietnam were 1.51 cancer/year/10^5^ individuals and 8 MoE, respectively, using a 20% prevalence rate of HBV [42]. Another study in Africa reported a cancer risk of 1.1 cancer/year/10^5^ people considering a 25% HBV prevalence rate and an MoE of 12.1 for Gambia [45]. The estimated mean cancer risk of 2.3 cancer/year/10^5^ people for the consumers was reported for Northern Vietnam, considering a 20% HBV prevalence rate, while, in adults, the value of cancer risk was 1.2 cancer/year/10^5^ people [43]. Because of the low contamination level in foods, the estimated dietary cancer risk in Japan is much lower as compared to our study results: even at the 99.9th percentile, it was 0.00059–0.00067 cancer/year/10^5^ population assuming a 1% HBV prevalence rate [72]. 

To reduce the synergistic effect of HBV and aflatoxins, the improvement in HBV vaccination as well as measures for the reduction of aflatoxin contamination in foods are necessary. The risk characterization of multiple-mycotoxins in rice will be useful for setting mycotoxin priority in adopting control measures. Moreover, the measured mycotoxin exposure due to rice in this study can be used in future total dietary exposure estimates in Pakistan, as the burden of cancer risk depends on the cumulative exposure. In addition to HBV and aflatoxins, Hepatitic C Virus (HCV) may also be taken into account in future coexposure studies, since it is one of the leading factors involved in HCC. Notably, HBV [54] and HCV [73] are highly prevalent in the Pakistani population.

## 4. Conclusions

The study measured the levels of multi-mycotoxin contamination in rice samples using a validated multi-mycotoxin LC–MS/MS method. The risk assessment of mycotoxins by rice intake in adults and children of the Pakistani population in SP and NP regions was performed after attaining consumption data of rice by conducting food frequency questionnaires. The mycotoxin contamination profile of Pakistani rice showed the prevalence of AFB_2_, AFB_1_, NIV, DAS, FB_1_, OTA, DON, HT2, and ZEN. Among the evaluated rice cooking recipes, Biryani demonstrated the highest degradation of AFB_2_ (63%) and AFB_1_ (51%). Our results indicate that dietary exposure to mycotoxins through rice was higher in children as compared to adults, and higher in NP in comparison to SP. The MoE of AFB_1_ was remarkably lower than the recommended safe limits. Moreover, there is a potential risk, due to rice consumption, of developing aflatoxin-induced hepatocellular carcinoma (HCC) in Pakistan. The Pakistani population is not exposed to a single mycotoxin but faces exposure to multiple mycotoxins. This study highlights the need to establish regulatory guidelines regarding the prevalence of mycotoxins in Pakistani foods, and a regular monitoring of highly consumed foodstuff, especially rice and cereals, is suggested. A cumulative risk assessment from the exposures of multi-mycotoxins, especially in the HBV- and HCV-infected population, also needs to be thoroughly studied in the future.

## 5. Materials and Methods 

### 5.1. Reagents and Chemicals 

Ochratoxin A (OTA = 10 µg/mL), aflatoxin mix (AFB_1_, AFB_2_, AFG_1_, AFG_2_ = 20 µg/mL), deoxynivalenol (DON = 100 µg/mL), zearalenone (ZEN = 100 µg/mL), Fumonisin mix (FB_1_, FB_2_ = 50 µg/mL), nivalenol (NIV = 100 µg/mL), neosolaniol (NEO = 100 µg/mL), deepoxy-deoxynivalenol (DOM = 50 µg/mL), T-2 toxin (T2 = 100 µg/mL), HT-2 toxin (HT2 = 100 µg/mL), 3-acetyldeoxynivalenol (3-ADON = 100 µg/mL), diacetoxyscirpenol (DAS = 100 µg/mL), 15-acetyldeoxynivalenol (15-ADON = 100 µg/mL), fusarenon X (FX = 100 µg/mL), and sterigmatocystin (STERIG = 50 µg/mL) were obtained as certified mycotoxin standard solutions in acetonitrile from Biopure (RomerLabs, Tulln, Austria). Fumonisin B_3_ (FB_3_) was obtained from Promec unit (Tygerberg, South Africa). Zearalanone (ZAN), alternariol (AOH) and alternariol monomethylether (AME) were purchased from Sigma and roquefortine C (ROQ-C) from Alexis Biochemicals (Enzo Life Sciences BVBA, Zandhoven, Belgium). The stock solution of FB_3_ (1 mg/mL) was made in acetonitrile/water (50/50, *v*/*v*). The stock solutions of AOH and AME (1 mg/mL) were prepared in methanol/dimethylformamide (60/40, *v*/*v*). ROQ-C and ZAN stock soution (1 mg/mL) were prepared in methanol. The stock solution of FB_3_ was stored at 4 °C, while all the others were stored at −20 °C for one year or until the expiration date. Working solutions were made by diluting the stock solutions in methanol and were stored at −20 °C for 3 months. A working solution of a standard mixture was prepared with the following concentrations: OTA, AFB_1_, AFB_2_, AFG_1_, and AFG_2_ (0.2 ng/µL); DAS (0.5 ng/µL); ROQ-C (1 ng/µL); 15-ADON (2.5 ng/µL); 3-ADON and STREG (5 ng/µL); ZEN, NEO, and AOH (10 ng/µL); T2-toxin and HT2-toxin (2.5 ng/uL); NIV, Fux-X, and AME (20 ng/µL); FB_3_ (25 ng/µL); DON, FB_1_, and FB_2_ (40 ng/µL).

### 5.2. Sampling and Food Consumption Data 

**Figure 2:**
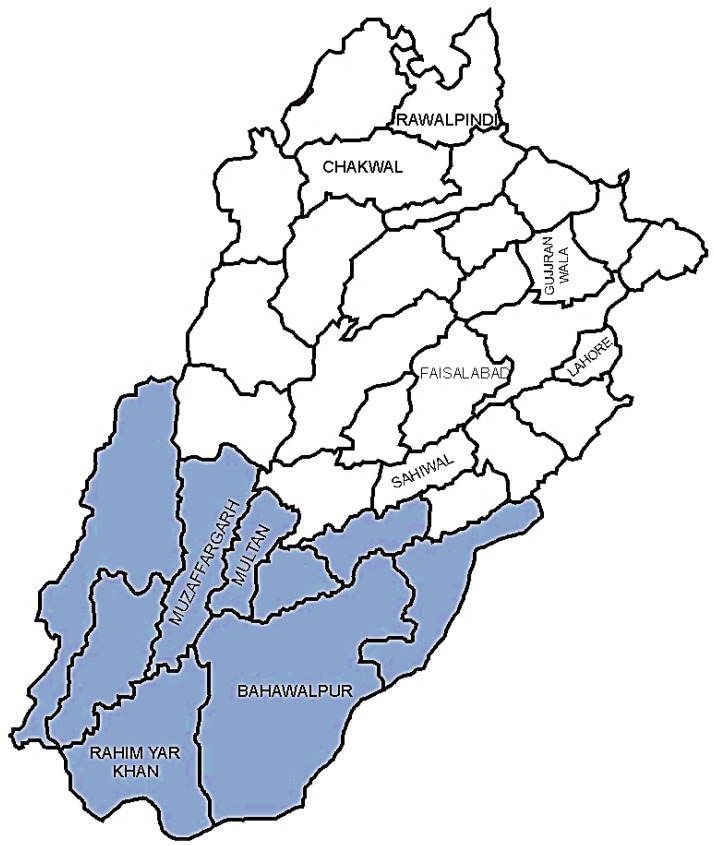
Map of Punjab province, Pakistan (30°00′ N 70°00′ E). The sampling points and districts are labelled. The colored region represents the southern irrigated zone (SP), while the white region shows the central-northern irrigated zone (NP).

Polished rice of all varieties and brands, intended for human consumption, was randomly purchased in the quantity of 1 kg from different wholesale markets, super markets, and small shops located in ten districts of two different agroecological zones of Punjab (North and South Punjab), Pakistan, during the year 2015. Ninety samples from each region were collected (Figure 2). The average rainfall in South Punjab (SP) and North Punjab (NP) are between 22.65 mm and 66.99 mm, respectively. The average annual temperatures in SP and NP are 26 °C and 24 °C, respectively. The location of SP and NP in coordinates (latitude and longitude) are between 28–30° N, 70–71° E, and 31–33° N, 72–74° E, respectively [74,75]. 

All samples were ground using an M 20-grinder (Ika-Werke, Staufen, Germany) and kept in plastic bags at −20 °C before mycotoxin determination. To obtain accurate exposure estimates, rice consumption data were obtained by conducting a survey in southern and northern Punjab regions of Pakistan. A FFQ was prepared, and individuals and families were interviewed. Portion-size pictures (small, medium, and large servings of cooked rice, i.e., 50 g, 75 g, 100 g of uncooked rice) were used to gather information on the rice intake, and the actual weight of each portion size was measured. The diet intake information for one week was gathered from the participants, and the mean daily rice intake of each individual was calculated (per kg of body weight per day). The proportions of participants from South Punjab (SP) and North Punjab (NP) regions in gathering the consumption data were 48% and 52%, respectively. The gender distribution of the participants was 48% male and 52% female. In total, 548 individuals in the adult category were interviewed, and the data of 467 individuals in the children category (age 7–15 years) was gathered by interviewing either the children or the female family head. Finally, the data from both regions was arranged separately for each category (adults and children) to get the mean, median, minimum, maximum, and percentile (P75, P90, P95) intake of rice. Furthermore, the generated consumption data was used in calculating the dietary exposure to mycotoxins.

### 5.3. Sample Preparation

The sample extraction methodology described by Monbaliu et al. [76] was followed. Internal standards were added to the samples before extraction. Five grams of the rice sample was extracted with 20 mL of acetonitrile/water/acetic acid (79/20/1, *v*/*v*/*v*) by agitating on a vertical shaker for 1 h and centrifuged for 15 min at 3300 *g*. The supernatant was subjected to cleanup by octadecyl (C_18_) solid phase extraction (SPE) column (Grace octadecyl C18, Lokeren, Belgium) on a vacuum elution manifold after conditioning with 10 mL of the extraction solvent—acetonitrile/water/acetic acid (79/20/1, *v*/*v*/*v*). The extraction was performed a second time by adding 5 mL of the extraction solvent to the samples. The eluate was collected in a 25 mL volumetric flask. The volume was adjusted with the extraction solvent. The extract was defatted with 10 mL *n*-hexane. Then the extract was split into two parts to perform two different modes of cleanup. In the first cleanup, 12.5 mL of the defatted extract was diluted with 27.5 mL of acetonitrile/acetic acid (99/1, *v*/*v*), and 30 mL of this extract was passed through Multisep226, Afla-ZON^+^ Multifunctional columns from Romers Lab. (Gernsheim, Germany), followed by washing with 5 mL of acetonitrile/acetic acid (99/1, *v*/*v*). In the second cleanup mode, the defatted extract (10 mL) was filtered through a Whatman glass microfilter (VWR International, Zaventem, Belgium), and 2 mL of this filtered extract was combined with the MultiSep 226 eluate. The combined eluates were evaporated, and the residue was dissolved in 150 µL of mobile phase containing methanol/water/acetic acid (57.2/41.8/1, *v*/*v*/*v*) and 5 mM ammonium acetate. Before LC–MS/MS analysis, the resulting solution was ultracentrifuged for 5 min at 14,000 *g* using ultra free-MC centrifugal filters (Bedford, MA, USA). 

### 5.4. Analysis by LC–MS/MS

A micromass Quatro Micro triple quadrupole mass spectrometer (Waters, Milford, MA, USA) equipped with a Waters Acquity UPLC system was used to analyze the samples, and the Masslynx (4.1) software (Micromass, Manchester, UK) was used for data processing. The analytical column was a Symmetry C18, 5 µm, 2.1 × 150 mm (Waters, Zellik, Belgium), with a guard column of Waters Sentry 3.5 µm 2.1 × 10 mm (Waters, Zellik, Belgium). The column and autosampler temperature was kept at 30 °C, and 20 µL was injected. Capillary voltage was set at 3.2 kV with a source voltage of 150 °C and a 350 °C desolvation temperature. Liquid chromatography conditions (mobile phase composition and gradient) and MS parameters were followed as described by Monbaliu et al. [77].

### 5.5. Quality Control and Quality Assurance

A set of performance characteristics that were in compliance with the recommendations defined by EU Commission Regulation EC/401/2006 was evaluated [49]. Deepoxy-deoxynivalenol (DOM) and zearalanone (ZAN), structural analogues of the type-B trichothecenes, and ZEN were used as internal standards in the multi-mycotoxin determination to compensate for matrix effects and for losses during extraction and cleanup. For each mycotoxin, a spiking experiment was performed at five concentration levels except for aflatoxins, which were spiked at six different levels each day in triplicate for four validation days (Table A1). The validation parameters assessed were: linearity, apparent recovery, limit of detection (LOD), limit of quantification (LOQ), intraday repeatability (RSD_r_), interday repeatability (RSD_R_), and expanded uncertainty. The linearity was tested graphically using a scatter plot, and the linear regression model was evaluated using a lack-of-fit test. The apparent recovery was calculated by dividing the observed value (quantified using calibration plot) by the spiked level. The sensitivity of the method was estimated by LOD. A series of blank rice samples spiked at low concentration levels were used to estimate LODs and LOQs, which provided a signal to noise ratio of 3:1 and 10:1 for the weakest transitions in LC–MS/MS chromatograms for each of the analyte, respectively. The precision in terms of intraday repeatability (the analysis of three replicates on the same day) and interday repeatability (the analysis of three replicates on four different days) was calculated using relative the standard deviation (RSD) at the spiked concentration levels (*n* = 5). The expanded measurement uncertainty (U) was obtained by multiplying the combined standard uncertainty (uc) by a coverage factor *k* = 2, based on the desired level of confidence of approximately 95%, where the uc was an estimated standard deviation calculated as the positive square root of the total variance obtained by combining the intralaboratory repeatability (sR), the uncertainty associated with the purity of the standards (U (Cref)), and the uncertainty associated with the mean recovery (sbias). 

### 5.6. Cooking of Rice by Pakistani Recipes

Three most common local cooking methods of rice in Pakistan were evaluated for their efficiency in the degradation of AFB_1_ and AFB_2_. Negative control samples were also washed and cooked as in each treatment, and spiked with known concentrations of standards after cooking. The aim was to make matrix-matched calibration curves and quantify the levels of AFB_1_ and AFB_2_ in the treated samples. The positive controls were samples of naturally contaminated uncooked rice that were used for comparison of the treatments to conclude the percentage of AFs degradation.

Naturally contaminated rice samples (100 g each in triplicate for each treatment) were washed with water (200 mL) three times, soaked in 200 mL of water for 20 min, and finally the water was completely removed. This washing step was similar in all treatments. For the first recipe (treatment) of boiled rice, the washed rice was added to boiling water (200 mL) and cooked for about 20 min. In the second recipe (pulao), first a curry (ingredients: oil, onion, ginger and garlic paste, tomato, boiled chicken, and salt and chili) was prepared, and 200 mL of water was added to the curry. On boiling, the washed rice was added to the mixture and cooked for 10 min at high flame and 20 min at low flame, while covering it tightly with a lid. For the third recipe (Biryani), the washed rice was boiled in excess water for 5 min, and the water was removed. The curry was prepared separately, having the same ingredients as those in pulao with additional yogurt and spices like cumin, pepper, cloves, cinnamon, cardamom, bay leaves, coriander, and mint leaves. Layers of curry and the boiled rice, alternatively on top of each other, were made in a pot and further cooked for 20 min at low flame after covering tightly with a lid. The cooked rice was cooled down and freeze dried. Then the samples were ground and analyzed for aflatoxin levels after sample preparation, following the methodology described by Majeed et al. [78]. 

### 5.7. Dietary Exposure Assessment 

The dietary exposure of mycotoxins was calculated by a deterministic risk analysis (Equation (1)).

Dietary Exposure = concentration of mycotoxin × daily rice intake per kg of body weight(1)

The left-censored mycotoxin contamination data related to the non-detects (ND), and those below the limit of quantification (<LOQ) can be a source of uncertainty in exposure models [79]. So, three different scenarios (lower bound, medium bound, and upper bound) were incorporated in this study to cope with the uncertainty, following the approach described by EFSA [80]. The dietary exposure levels were estimated considering two approaches, using a fixed mycotoxin concentration and variable values (mean, median, maximum, and probability values) of consumption level, and using fixed consumption levels with variable values (mean, median, maximum, and probability values) of mycotoxin levels. In each approach, all three scenarios were considered: Upper bound (<LOQ = LOQ), Medium bound (<LOQ = ½ LOQ), and lower bound (<LOQ = 0).

### 5.8. Risk Characterization 

The risk characterization of the genotoxic aflatoxins was performed by both margin of exposure MoE [23] and cancer risk approaches [22]. The MoE was estimated (Equation (2)) by the ratio of Bench Mark Dose Level (BMDL) that causes a 10% increase in the cancer incidence in rodents (BMDL_10_ = 170 ng/kg b.w. day) and the exposure to AFB_1_ [23].

Margin of exposure = BMDL_10_/exposure (ng/kg b.w. day) (2)

The risk of AFB_1_-induced cancer (hepatocellular carcinoma, HCC) was calculated (Equation (3)) by multiplying the probability of cancer with the AFB_1_ exposure estimates of min, max, mean, and percentiles in both upper and lower bound scenarios for each category in both regions. Here, cancer potency P_cancer_ (Equation (4)) deals with the percentage of both carriers (%Pop.HBsAg^+^ = 0.024) and noncarriers (%Pop.HBsAg^−^ = 0.976) of HBV infection in the Pakistani population, that is 2.4% [54], as well as with the carcinogenic potency of AFB_1_ for carriers (P_HBsAg+_ = 0.3 cancer/year/10^5^ individuals) and noncarriers (P_HBsAg-_ = 0.01 cancer/year/10^5^ individuals).

Cancer risk = P_cancer_ × Exposure (ng/kg b.w. day) (3)

while

P_cancer_ = (P_HBsAg+_ × %Pop.HBsAg^+^) + (P_HBsAg−_ × %Pop.HBsAg^−^). (4)

### 5.9. Statistical Analysis

The normality of the consumption data distribution and contamination data was assessed by Kolmogorov–Smirnov, Shapiro–Wilk test, and the corresponding Q/Q plots. A non-parametric Mann–Whitney U test was applied to determine the significance, using the SPSS statistical package (IBM^®^, Version 14, SPSS Inc. Chicago, IL, USA, 2005) with a level of confidence of 0.05. All other calculations were executed in Excel 2010. The mean data together with standard deviations (SD) was stated. Significant differences in percentage degradation in AFB_1_ and AFB_2_ among various cooking processes were determined by Tukey’s HSD test (IBM^®^, Version 14, SPSS Inc. Chicago, IL, USA, 2005).

## Figures and Tables

**Table 1:** Limits of detection (LOD) and limits of quantification (LOQ), linearity range, and coefficient of determination (R^2^) of all the measured mycotoxins in rice.

Mycotoxin	LOD (μg/kg)	LOQ (μg/kg)	Range (μg/kg)	*R*^2^
Zearalenone (ZEN)	7	13	37.5–150	0.998
Enniatin B (ENNB)	50	100	100–400	0.992
Roquefortine-C (ROQ-C)	4	8	10–40	0.993
Sterigmatocystine (STREG)	9	18	25–100	0.995
Alternariol methylether (AME)	11	21	100–400	0.991
Fumonisin B_2_ (FB_2_)	17	35	100–400	0.993
Ochratoxin A (OTA)	0.75	2.5	2.5–20	0.994
Fumonisin B_3_ (FB_3_)	53	105	125–500	0.995
T-2 toxin (T2)	22	44	50–200	0.993
Fumonisin B_1_ (FB_1_)	13	26	100–400	0.994
HT-2 toxin (HT2)	13	26	50–200	0.996
Alternariol (AOH)	22	44	50–200	0.997
Diacetoxyscirpenol (DAS)	3	6	10–40	0.996
Aflatoxin B_1_ (AFB_1_)	0.5	1.5	2.5–20	0.994
Aflatoxin B_2_ (AFB_2_)	0.5	1.5	2.5–20	0.995
Aflatoxin G_1_ (AFG_1_)	0.5	1.5	2.5–20	0.996
Aflatoxin G_2_ (AFG_2_)	0.5	1.5	2.5–20	0.994
15-Acetyl-deoxynivalenol (15-ADON)	9	19	25–100	0.995
3-Acetyl-deoxynivalenol (3-ADON)	16	33	50–200	0.998
Fusarenon-X (FX)	9	18	100–400	0.993
Neosolaniol (NEO)	12	23	50–200	0.996
Deoxynivalenol (DON)	19	37	100–400	0.992
Nivalenol (NIV)	8	16	50–200	0.999

**Table 2:** Contamination levels (µg/kg) and incidence rate (%) of different mycotoxins in all rice samples (*n* = 180).

Descriptive Statistics	AFB_2_	AFB_1_	AFs	NIV	DAS	FB_1_	OTA	DON	HT2	ZEN
Mean	1.91	5.84	7.75	13.8	1.60	22.9	0.610	6.99	2.03	8.48
Median	<LOD	5.00	7.00	<LOD	<LOD	<LOD	<LOD	<LOD	<LOD	<LOD
Stdev.	2.42	6.69	8.75	28.2	2.92	28.8	2.68	22.7	7.26	21.57
Min.	<LOD	<LOD	<LOD	<LOD	<LOD	<LOD	<LOD	<LOD	<LOD	<LOD
Max.	9.15	40.0	44.1	116	12.53	75.1	24.0	115	32.0	114
P75	2.80	8.65	12.2	16.0	0.00	58.2	<LOD	<LOD	<LOD	<LOD
P90	5.20	15.1	21.2	55.3	6.79	62.4	<LOD	<LOD	<LOD	47.7
P95	7.99	17.5	25.7	82.6	6.81	63.3	6.80	77.0	26.7	48.9
P99	8.40	23.2	30.7	116	7.52	69.0	11.0	81.5	30.2	114
% freq.	48	56	56	28	23	42	6.0	8.0	10	15

**Table 3:** Consumption data (mean ± SD g/individual/day) and intake (mean ± SD g/kg b.w. per day) of rice by children and adults in different regions of Pakistan and at national level (mean) obtained during the survey in 2015–2016.

Regions	Adults		Children	
	Daily Intake (g) Per Capita	Daily Intake (g) per kg Body Weight	Daily Intake (g) per Capita	Daily Intake (g) per kg Body Weight
South Punjab	91.3 ± 24.1 ^a^	1.66 ± 0.508 ^a^	50.8 ± 11.8 ^a^	1.68 ± 0.513 ^a^
North Punjab	124 ± 24.0 ^a^	2.18 ± 0.739 ^a^	62.5 ± 18.8 ^a^	2.26 ± 0.726 ^a^
Mean (national level)	108 ± 24.1	1.92 ± 0.624	56.6 ± 15.3	1.97 ± 0.619

^a^ Values with the same alphabet letters are not significantly different (*p* < 0.05).

**Table 4:** Percentage of degradation of aflatoxin B_1_ and B_2_ in rice after washing and with different methods of cooking.

Treatments	% Degradation AFB_1_	% Degradation AFB_2_
Control	0.0 ^d^	0.0 ^d^
Washing with water	14 ± 1.1 ^c^	15 ± 1.1 ^c^
Pulao (Pakistani Recipe)	41 ± 1.2 ^b^	47 ± 1.5 ^b^
Biryani (Pakistani Recipe)	51 ± 2.0 ^a^	63 ± 2.3 ^a^
Boiled rice	42 ± 1.4 ^b^	50 ± 1.7 ^b^
On average	45 ± 1.4	53 ± 1.7

Values are the mean of three replicates. Values with different alphabet letters (^a, b, c, d^) indicate significant differences in percentage degradation in aflatoxin B_1_ and aflatoxin B_2_ among various cooking processes, as determined by Tukey’s HSD test.

**Table 5:** Deterministic dietary exposures (ng/kg b.w./day) through the consumption of rice contaminated by multiple mycotoxins by the adult group of the population in North and South Punjab of Pakistan, using the medium bound scenario (<LOQ = ½ LOQ). The calculations are based on (**A**) fixed mean mycotoxin concentration and variable rice consumption in the two regions and (**B**) fixed mean rice consumption and variable mycotoxins concentration in the two regions. Values exceeding the PMTDI (provisional maximum tolerable daily intake) are shown in bold.

**(A) Deterministic Exposure Analysis at Fixed Mycotoxin Concentration and Variable Rice Consumption.**																		
**Descriptive Statistics**	**AFB_2_**		**AFB_1_**		**NIV**		**DAS**		**FB_1_**		**OTA**		**DON**		**HT2**		**ZEN**	
	**SP**	**NP**	**SP**	**NP**	**SP**	**NP**	**SP**	**NP**	**SP**	**NP**	**SP**	**NP**	**SP**	**NP**	**SP**	**NP**	**SP**	**NP**
Mean	**1.08**	**1.76**	**4.11**	**7.21**	21.9	39.0	3.15	4.50	32.5	60.7	0.92	1.68	10.8	16.6	5.29	9.50	15.4	23.9
Median	**1.02**	**1.65**	**3.89**	**6.75**	20.7	36.5	2.98	4.21	30.7	56.8	0.87	1.57	10.2	15.5	5.01	8.89	14.5	22.3
Min.	**0.660**	**0.871**	**2.50**	**3.54**	13.3	19.2	1.92	2.21	19.7	29.8	0.56	0.82	6.58	8.15	3.22	4.67	9.34	11.7
P75	**1.21**	**2.02**	**4.61**	**8.27**	24.6	44.7	3.54	5.15	36.5	69.5	1.03	1.92	12.2	19.0	5.94	10.9	17.3	27.3
P90	**1.62**	**2.95**	**6.18**	**12.1**	32.9	65.2	4.74	7.52	48.8	101	1.38	2.81	16.3	27.8	7.96	15.9	23.1	39.8
P95	**1.75**	**3.02**	**6.65**	**12.4**	35.4	66.9	5.10	7.71	52.5	104	1.49	2.88	17.5	28.5	8.56	16.3	24.8	40.9
Max.	**2.48**	**4.15**	**9.44**	**17.0**	50.2	91.9	7.24	10.6	74.6	143	2.12	3.96	24.8	39.1	12.2	22.4	35.3	56.3
**(B) Deterministic Exposure Analysis at Fixed Rice Consumption and Variable Mycotoxin Concentration.**																		
**Descriptive Statistics**	**AFB_2_**		**AFB_1_**		**NIV**		**DAS**		**FB_1_**		**OTA**		**DON**		**HT2**		**ZEN**	
	**SP**	**NP**	**SP**	**NP**	**SP**	**NP**	**SP**	**NP**	**SP**	**NP**	**SP**	**NP**	**SP**	**NP**	**SP**	**NP**	**SP**	**NP**
Mean	**1.08**	**1.76**	**4.11**	**7.21**	21.9	39.0	3.15	4.50	32.5	60.6	0.920	1.68	10.8	16.6	5.29	9.50	15.4	23.9
Median	**0.310**	**1.61**	**4.07**	**5.40**	<LOD	<LOD	<LOD	<LOD	<LOD	14.2	<LOD	<LOD	<LOD	<LOD	<LOD	<LOD	<LOD	<LOD
Min.	<LOD	<LOD	<LOD	<LOD	<LOD	<LOD	<LOD	<LOD	<LOD	<LOD	<LOD	<LOD	<LOD	<LOD	<LOD	<LOD	<LOD	<LOD
P75	**1.71**	**2.91**	**5.86**	**10.4**	13.3	39.3	4.98	8.24	97.0	127	<LOD	<LOD	<LOD	<LOD	<LOD	<LOD	11.6	15.3
P90	**2.66**	**4.38**	**10.9**	**16.3**	90.2	133.8	11.3	14.8	97.0	139	1.24	1.64	<LOD	<LOD	21.6	32.3	78.3	104
P95	**4.39**	**6.69**	**13.3**	**19.0**	124	192	11.3	14.9	104	144	4.88	15.1	124	168	21.6	58.68	79.5	111
Max.	**5.16**	**7.39**	**14.6**	**42.8**	177	254.	20.8	16.4	105	164	**18.3**	**52.4**	127	251	51.4	69.83	190	**251**

PMTDI values (ng/kg b.w. day): AFB_1_ = 0.0001, AFB_2_ = 0.0001, NIV = 700, DAS = 100, FB_1_ = 2000, OTA= 17, DON = 1000, HT2 = 100, ZEN = 250.

**Table 6:** Deterministic dietary exposures (ng/kg b.w./day) through the consumption of rice contaminated by multiple mycotoxins by children in North and South Punjab of Pakistan, using the medium bound scenario (<LOQ = ½ LOQ). The calculations are based on (**A**) fixed mean mycotoxin concentration and variable rice consumption in the two regions and (**B**) fixed mean rice consumption and variable mycotoxins concentration in the two regions. Values exceeding the PMTDI (provisional maximum tolerable daily intake) are shown in bold.

**(A) Deterministic Exposure Analysis at Fixed Mycotoxin Concentration and Variable Rice Consumption.**																		
**Descriptive Statistics**	**AFB_2_**		**AFB_1_**		**NIV**		**DAS**		**FB_1_**		**OTA**		**DON**		**HT2**		**ZEN**	
	**SP**	**NP**	**SP**	**NP**	**SP**	**NP**	**SP**	**NP**	**SP**	**NP**	**SP**	**NP**	**SP**	**NP**	**SP**	**NP**	**SP**	**NP**
Mean	**1.09**	**1.83**	**4.16**	**7.48**	22.1	40.5	3.19	4.67	32.8	62.9	0.931	1.74	10.9	17.2	5.36	9.86	15.5	24.7
Median	**1.00**	**1.73**	**3.79**	**7.08**	20.2	38.3	2.91	4.42	29.9	59.6	0.850	1.65	9.98	16.3	4.88	9.33	14.2	23.4
Min.	**0.720**	**0.991**	**2.75**	**4.05**	14.6	21.9	2.11	2.52	21.7	34.1	0.620	0.941	7.23	9.32	3.54	5.33	10.3	13.4
P75	**1.16**	**2.16**	**4.42**	**8.86**	23.5	47.9	3.39	5.52	34.9	74.5	0.990	2.06	11.6	20.4	5.71	11.6	16.5	29.3
P90	**1.39**	**2.6**	**5.31**	**10.6**	28.2	57.5	4.07	6.63	41.9	89.4	1.19	2.47	13.9	24.5	6.84	14.1	19.8	35.2
P95	**1.86**	**2.88**	**7.08**	**11.8**	37.7	63.8	5.43	7.36	55.9	99.3	1.59	2.75	18.6	27.2	9.12	15.6	26.5	39.1
Max.	**2.79**	**3.93**	**10.6**	**16.1**	56.5	87.1	8.14	10.0	83.8	135	2.38	3.75	27.9	37.1	13.7	21.2	39.7	53.3
**(B) Deterministic Exposure Analysis at Fixed Rice Consumption and Variable Mycotoxin Concentration.**																		
**Descriptive Statistics**	**AFB_2_**		**AFB_1_**		**NIV**		**DAS**		**FB_1_**		**OTA**		**DON**		**HT2**		**ZEN**	
	**SP**	**NP**	**SP**	**NP**	**SP**	**NP**	**SP**	**NP**	**SP**	**NP**	**SP**	**NP**	**SP**	**NP**	**SP**	**NP**	**SP**	**NP**
Mean	**1.09**	**1.83**	**4.16**	**7.48**	22.1	40.5	3.19	4.67	32.9	62.9	0.931	1.74	10.9	17.2	5.36	9.86	15.6	24.7
Median	**0.311**	**1.67**	**4.11**	**5.60**	<LOD	<LOD	<LOD	<LOD	<LOD	14.7	<LOD	<LOD	<LOD	<LOD	<LOD	<LOD	<LOD	<LOD
Min.	<LOD	<LOD	<LOD	<LOD	<LOD	<LOD	<LOD	<LOD	<LOD	<LOD	<LOD	<LOD	<LOD	<LOD	<LOD	<LOD	<LOD	<LOD
P75	**1.73**	**3.01**	**5.93**	**10.8**	13.4	40.7	5.04	8.54	97.8	131	<LOD	<LOD	<LOD	<LOD	<LOD	<LOD	11.7	15.8
P90	**2.71**	**4.54**	**11.1**	**16.9**	91.3	138	11.4	15.4	97.8	143	1.26	1.70	<LOD	<LOD	21.8	33.5	79.3	108
P95	**4.44**	**6.94**	**13.5**	**19.7**	125	199	11.5	15.4	105	149	4.93	15.6	125	174	21.8	60.9	80.5	116
Max.	**5.22**	**7.66**	**14.8**	**44.4**	179	264	21.0	17.0	106	170	**18.5**	**54.3**	129	260	52.1	72.4	192	**259**

PMTDI values (ng/kg b.w. day): AFB_1_ = 0.0001, AFB_2_ = 0.0001, NIV = 700, DAS = 100, FB_1_ = 2000, OTA = 17, DON = 1000, HT2 = 100, ZEN = 250.

**Table 7:** Estimation of the Margin of Exposure in children and adults of South and North Punjab, Pakistan, using variable consumption data and a fixed mean AFB_1_ concentration considering both lower bound (LB) and upper bound (UB) scenarios.

**Deterministic Analysis**	**Descriptive Levels**	**South Punjab**				**North Punjab**			
		**Adult**		**Children**		**Adult**		**Children**	
		**UB**	**LB**	**UB**	**LB**	**UB**	**LB**	**UB**	**LB**
Fixed Mycotoxin concentration	Mean	38.0	41.7	37.6	41.2	23.3	23.7	22.4	22.9
	Median	40.2	44.1	41.2	45.2	24.9	25.4	23.7	24.2
	Min.	62.6	68.7	56.9	62.5	47.4	48.3	41.5	42.3
	P75	33.9	37.2	35.3	38.8	20.3	20.7	19.0	19.3
	P90	25.3	27.8	29.4	32.3	13.9	14.2	15.8	16.1
	P95	23.5	25.8	22.1	24.2	13.6	13.8	14.2	14.5
	Max.	16.6	18.2	14.7	16.2	9.9	10.1	10.4	10.6

**Table 8:** Estimation of cancer risk in children and adults of South and North Punjab, Pakistan, using exposures at medium bound values of both scenarios (fixed mean AFB_1_ concentration with variable consumption data and fixed consumption data with variable AFB_1_ concentration).

Deterministic Analysis	Descriptive Levels	South Punjab		North Punjab	
		Adult	Children	Adult	Children
A. At fixed Mycotoxin Concentration	Mean	0.070	0.071	0.122	0.127
	Median	0.066	0.064	0.114	0.120
	Min.	0.042	0.047	0.060	0.069
	P75	0.078	0.075	0.140	0.150
	P90	0.105	0.090	0.205	0.180
	P95	0.113	0.120	0.210	0.200
	Max.	0.160	0.180	0.288	0.273
B. At fixed Consumption of Rice	Mean	0.070	0.071	0.122	0.127
	Median	0.069	0.070	0.092	0.095
	Min.	<LOD	<LOD	<LOD	<LOD
	P75	0.099	0.101	0.177	0.184
	P90	0.186	0.188	0.276	0.287
	P95	0.226	0.229	0.322	0.334
	Max.	0.248	0.251	0.725	0.752

## Appendix A

**Table A1:** Intraday repeatability (RSDr), interday repeatability (RSDR), apparent recovery (%), and expanded measurement uncertainty (U) of the individual mycotoxins for the rice matrix analyzed at spiked concentration levels, in triplicate on four different days.

Mycotoxin	Spike Conc. (µg/kg)	RSD_r_ (%)	RSD_R_ (%)	Apparent Recovery (%)	Expanded Measurement Uncertainty U (%) *
ZEN	37.5	8	14	100	6
	56.3	4	16	99	3
	75	6	12	100	5
	100	5	9	100	3
	150	4	7	100	2
ENNB	100	14	20	80	17
	150	12	17	86	17
	200	9	17	111	13
	300	12	14	96	24
	400	10	12	99	22
ROQ-C	10	9	16	111	34
	15	8	14	99	19
	20	5	14	93	14
	30	3	12	98	3
	40	3	8	102	4
STREG	25	13	19	110	16
	37.5	11	17	97	12
	50	8	14	94	8
	75	5	15	99	5
	100	4	12	101	3
AME	100	11	12	102	9
	150	9	12	99	4
	200	6	11	99	3
	300	7	10	99	3
	400	5	9	100	3
FB_2_	100	9	16	98	5
	150	7	13	100	3
	200	6	11	100	4
	300	6	9	99	2
	400	5	8	100	2
OTA	2.5	14	19	101	14
	5	11	17	99	5
	10	9	13	100	11
	15	8	14	99	4
	20	5	11	100	2
FB_3_	125	7	16	99	3
	187.5	8	14	99	3
	250	6	11	100	4
	375.5	5	8	99	3
	500	4	7	99	3
T2	50	13	18	106	30
	75	10	15	96.	17
	100	11	14	99.	9
	150	11	14	99.	7
	200	9	13	100	6
FB_1_	100	9	18	99	3
	150	8	16	100	2
	200	5	12	100	3
	300	4	9	99	3
	400	4	8	100	2
HT2	50	14	20	105	26
	75	13	19	98	19
	100	9	17	97	7
	150	6	13	100	3
	200	3	12	100	4
AOH	50	9	19	101	18
	75	9	17	99	17
	100	7	15	98	16
	150	6	11	101	14
	200	3	8	99	9
DAS	10	7	16	98	21
	15	5	15	102	17
	20	6	11	99	11
	30	6	9	99	8
	40	4	7	100	4
AFB_1_	2.5	8	15	95	16
	5	7	13	100	16
	7.5	7	11	96	11
	10	5	9	100	9
	15	4	7	102	7
	20	4	7	98	4
AFB_2_	2.5	7	17	96	14
	5	7	15	100	12
	7.5	5	13	97	11
	10	5	12	101	5
	15	4	9	100	3
	20	3	7	99	4
AFG_1_	2.5	7	18	98	13
	5	6	18	101	12
	7.5	5	15	99	11
	10	4	12	101	7
	15	3	9	98	7
	20	3	6	100	5
AFG_2_	2.5	8	16	97	14
	5	7	15	101	13
	7.5	6	13	99	11
	10	5	13	100	7
	15	5	8	99	5
	20	4	7	100	4
15-ADON	25	13	15	86	7
	37.5	11	11	84	6
	50	8	11	84	5
	75	6	9	85	3
	100	4	9	85	3
3-ADON	50	12	20	102	9
	75	11	16	99	7
	100	9	16	98	6
	150	6	14	100	3
	200	5	11	100	2
FX	100	13	19	100	8
	150	11	17	100	5
	200	9	17	99	4
	300	7	13	99	3
	400	5	12	100	3
NEO	50	9	17	100	9
	75	7	17	99	7
	100	6	15	100	6
	150	4	11	99	4
	200	5	9	100	3
DON	100	7	15	102	8
	150	8	13	99	6
	200	6	10	99	5
	300	5	9	100	4
	400	3	6	100	3
NIV	50	7	16	99	5
	75	6	14	99	4
	100	7	13	100	3
	150	5	11	99	3
	200	4	8	100	3

* With a maximum error limit of 20% and a β-tolerance interval of 0.85. Abbreviation: ZEN = zearalenone; ENNB = Enniatin B; ROQ-C = roquefortine-C; STERIG = sterigmatocystine; AME = alternariol methylether; FB_1_, FB_2_, and FB_3_ = fumonisin B_1_, B_2_, and B_3_; OTA = ochratoxin A; T2 = T-2 toxin; HT2 = HT-2 toxin; AOH = Alternariol; DAS = diacetoxyscirpenol; AFB_1_, B_2_, G_1_, and G_2_ = aflatoxin B_1_, B_2_, G_1_ and G_2_: 15-ADON = 15-acetyl-deoxynivalenol; 3-ADON = 3-acetyl-deoxynivalenol; F-X = fusarenon-X; NEO = neosolaniol; DON = deoxynivalenol; NIV = nivalenol.

## Appendix B

Survey Form: Food Frequency Questionnaires

Interview #____

**Date of Interview**	
Region/Province	
City	

**Name**	**Age (√)**		**Sex (√)**		**Weight (kg)**
	**7–15 Years**	**16–65 Years**	**Male**	**Female**	
					

**Storage Time of the Rice**	
Variety of rice you use	
Typical Recipes of rice How many times (n) you eat in one week	Biryani:
	Polao:
	Boil rice:
	Other: (please name)
Portion Size	Plate (serving size) (S: small, M: medium, L: large)

**Frequency of Rice Consumption during One Week**			
Serving Size (√) and Number (n)	Small	Medium	Large
**Monday**			
Breakfast			
Lunch			
Dinner			
**Tuesday**			
Breakfast			
Lunch			
Dinner			
**Wednesday**			
Breakfast			
Lunch			
Dinner			
**Thursday**			
Breakfast			
Lunch			
Dinner			
**Friday**			
Breakfast			
Lunch			
Dinner			
**Saturday**			
Breakfast			
Lunch			
Dinner			
**Sunday**			
Breakfast			
Lunch			
Dinner			

## Appendix C

**Table A2:** Deterministic dietary exposures (ng/kg b.w./day) through the consumption of rice contaminated with multiple mycotoxins by the adult group of the population in North and South Punjab of Pakistan, using the upper bound scenario (<LOQ = LOQ). The calculations are based on (**A**) fixed mean mycotoxin concentration and variable rice consumption in the two regions and (**B**) fixed mean rice consumption and variable mycotoxins concentration in the two regions. Values exceeding the PMTDI (provisional maximum tolerable daily intake) are shown in bold.

**(A) Deterministic Exposure Analysis at Fixed Mycotoxin Concentration and Variable Rice Consumption.**																		
**Descriptive Statistics**	**AFB_2_**		**AFB_1_**		**NIV**		**DAS**		**FB_1_**		**OTA**		**DON**		**HT2**		**ZEN**	
	**SP**	**NP**	**SP**	**NP**	**SP**	**NP**	**SP**	**NP**	**SP**	**NP**	**SP**	**NP**	**SP**	**NP**	**SP**	**NP**	**SP**	**NP**
Mean	**1.22**	**1.83**	**4.47**	**7.31**	25.1	42.3	3.84	5.35	34.1	61.9	1.17	1.93	10.8	17.0	8.51	12.9	17.2	26.7
Median	**1.16**	**1.71**	**4.23**	**6.83**	23.7	39.5	3.63	5.01	32.3	57.9	1.11	1.80	10.2	15.9	8.05	12.1	16.3	24.9
Min.	**0.741**	**0.901**	**2.72**	**3.59**	15.3	20.8	2.33	2.63	20.7	30.4	0.71	0.95	6.58	8.36	5.17	6.33	10.5	13.1
P75	**1.37**	**2.10**	**5.02**	**8.37**	28.2	48.4	4.31	6.13	38.3	70.9	1.31	2.21	12.2	19.5	9.55	14.8	19.3	30.6
P90	**1.84**	**3.06**	**6.72**	**12.2**	37.7	70.6	5.77	8.95	51.2	103	1.76	3.22	16.3	28.5	12.7	21.5	25.8	44.6
P95	**1.98**	**3.14**	**7.23**	**12.5**	40.6	72.4	6.21	9.18	55.1	106	1.89	3.31	17.5	29.2	13.7	22.1	27.8	45.8
Max.	**2.81**	**4.32**	**10.3**	**17.2**	57.6	99.6	8.82	12.6	78.2	145	2.68	4.54	24.8	40.1	19.5	30.4	39.5	62.9
**(B) Deterministic Exposure Analysis at Fixed Rice Consumption and Variable Mycotoxin Concentration.**																		
**Descriptive Statistics**	**AFB_2_**		**AFB_1_**		**NIV**		**DAS**		**FB_1_**		**OTA**		**DON**		**HT2**		**ZEN**	
	**SP**	**NP**	**SP**	**NP**	**SP**	**NP**	**SP**	**NP**	**SP**	**NP**	**SP**	**NP**	**SP**	**NP**	**SP**	**NP**	**SP**	**NP**
Mean	**1.22**	**1.83**	**4.47**	**7.31**	25.1	42.2	3.84	5.35	34.1	61.9	1.17	1.93	10.82	17.03	8.51	12.9	17.2	26.7
Median	**0.920**	**1.61**	**4.07**	**5.40**	<LOD	<LOD	<LOD	<LOD	<LOD	28.3	<LOD	<LOD	<LOD	<LOD	<LOD	<LOD	<LOD	<LOD
Min.	<LOD	<LOD	<LOD	<LOD	<LOD	<LOD	<LOD	<LOD	<LOD	<LOD	<LOD	<LOD	<LOD	<LOD	<LOD	<LOD	<LOD	<LOD
P75	**1.71**	**2.90**	**5.86**	**10.4**	26.5	39.3	9.96	13.1	96.6	127	<LOD	<LOD	<LOD	<LOD	<LOD	<LOD	21.6	28.4
P90	**3.19**	**4.38**	**12.9**	**16.3**	90.2	133	11.2	14.8	96.6	138	4.15	5.46	<LOD	<LOD	43.2	56.7	78.3	104
P95	**5.04**	**6.69**	**14.2**	**19.0**	124.3	192	11.3	14.8	103	144	6.76	15.1	124	168	43.2	58.7	79.5	111
Max.	**5.16**	**7.39**	**16.5**	**42.7**	177	254	20.7	16.4	105	163	**18.3**	**52.3**	127	250	51.5	69.8	190	**250**

PMTDI values (ng/kg b.w. day): AFB_1_ = 0.0001, AFB_2_ = 0.0001, NIV = 700, DAS = 100, FB_1_ = 2000, OTA = 17, DON = 1000, HT2 = 100, ZEN = 250.

## Appendix D

**Table A3:** Deterministic dietary exposures (ng/kg b.w./day) through the consumption of rice contaminated with multiple mycotoxins by children in North and South Punjab of Pakistan, using the upper bound scenario (<LOQ = LOQ). The calculations are based on (**A**) fixed mean mycotoxin concentration and variable rice consumption in the two regions and (**B**) fixed mean rice consumption and variable mycotoxins concentration in the two regions. Values exceeding the PMTDI (provisional maximum tolerable daily intake) are shown in bold.

**(A) Deterministic Exposure Analysis at Fixed Mycotoxin Concentration and Variable Rice Consumption.**																		
**Descriptive Statistics**	**AFB_2_**		**AFB_1_**		**NIV**		**DAS**		**FB_1_**		**OTA**		**DON**		**HT2**		**ZEN**	
	**SP**	**NP**	**SP**	**NP**	**SP**	**NP**	**SP**	**NP**	**SP**	**NP**	**SP**	**NP**	**SP**	**NP**	**SP**	**NP**	**SP**	**NP**
Mean	**1.24**	**1.90**	**4.53**	**7.58**	25.4	43.8	3.89	5.55	34.5	64.2	1.18	2.00	10.9	17.6	8.61	13.4	17.4	27.7
Median	**1.13**	**1.80**	**4.13**	**7.17**	23.2	41.5	3.54	5.26	31.4	60.7	1.08	1.89	9.98	16.7	7.85	12.7	15.8	26.2
Min.	**0.820**	**1.03**	**2.99**	**4.10**	16.7	23.7	2.57	3.00	22.7	34.7	0.780	1.08	7.23	9.56	5.68	7.24	11.5	14.9
P75	**1.32**	**2.25**	**4.81**	**8.97**	27.0	51.8	4.13	6.57	36.6	75.9	1.26	2.37	11.6	20.9	9.15	15.8	18.5	32.7
P90	**1.58**	**2.70**	**5.78**	**10.7**	32.4	62.2	4.96	7.88	44.0	91.1	1.51	2.84	13.9	25.1	10.9	18.9	22.2	39.3
P95	**2.11**	**3.00**	**7.70**	**11.9**	43.2	69.1	6.61	8.76	58.6	101.3	2.01	3.16	18.6	27.8	14.6	21.1	29.6	43.7
Max.	**3.16**	**4.09**	**11.5**	**16.3**	64.9	94.3	9.92	11.9	88.0	138	3.02	4.30	27.9	38.0	21.9	28.7	44.5	59.6
**(B) Deterministic Exposure Analysis at Fixed Rice Consumption and Variable Mycotoxin Concentration.**																		
**Descriptive Statistics**	**AFB_2_**		**AFB_1_**		**NIV**		**DAS**		**FB_1_**		**OTA**		**DON**		**HT2**		**ZEN**	
	**SP**	**NP**	**SP**	**NP**	**SP**	**NP**	**SP**	**NP**	**SP**	**NP**	**SP**	**NP**	**SP**	**NP**	**SP**	**NP**	**SP**	**NP**
Mean	**1.24**	**1.90**	**4.53**	**7.58**	25.4	43.8	3.89	5.55	34.5	64.2	1.18	2.00	10.9	17.6	8.61	13.4	17.4	27.7
Median	**0.930**	**1.67**	**4.11**	**5.60**	<LOD	<LOD	<LOD	<LOD	<LOD	29.4	<LOD	<LOD	<LOD	<LOD	<LOD	<LOD	<LOD	<LOD
Min.	<LOD	<LOD	<LOD	<LOD	<LOD	<LOD	<LOD	<LOD	<LOD	<LOD	<LOD	<LOD	<LOD	<LOD	<LOD	<LOD	<LOD	<LOD
P75	**1.73**	**3.00**	**5.93**	**10.8**	26.8	40.7	10.1	13.6	97.8	131	<LOD	<LOD	<LOD	<LOD	<LOD	<LOD	21.83	29.42
P90	**3.23**	**4.54**	**13.1**	**16.9**	91.2	138	11.4	15.3	97.8	143	4.20	5.66	0.00	0.00	43.6	58.8	79.2	108
P95	**5.10**	**6.94**	**14.4**	**19.7**	125	199	11.5	15.4	105	149	6.84	15.6	125	174	43.6	60.8	80.4	116
Max.	**5.22**	**7.66**	**16.8**	**44.4**	179	264	21.0	17.0	106	170	**18.5**	**54.3**	129	260	52.1	72.4	192	**259**

PMTDI values (ng/kg b.w. day): AFB_1_ = 0.0001, AFB_2_ = 0.0001, NIV = 700, DAS = 100, FB_1_ = 2000, OTA = 17, DON = 1000, HT2 = 100, ZEN = 250.

## Appendix E

**Table A4:** Deterministic dietary exposures (ng/kg b.w./day) through the consumption of rice contaminated with multiple mycotoxins by the adult group of the population in North and South Punjab of Pakistan, using the lower bound scenario (<LOQ = 0). The calculations are based on (**A**) fixed mean mycotoxin concentration and variable rice consumption in the two regions and (**B**) fixed mean rice consumption and variable mycotoxins concentration in the two regions. Values exceeding the PMTDI (provisional maximum tolerable daily intake) are shown in bold.

**(A) Deterministic Exposure Analysis at Fixed Mycotoxin Concentration and Variable Rice Consumption.**																		
**Descriptive Statistics**	**AFB_2_**		**AFB_1_**		**NIV**		**DAS**		**FB_1_**		**OTA**		**DON**		**HT2**		**ZEN**	
	**SP**	**NP**	**SP**	**NP**	**SP**	**NP**	**SP**	**NP**	**SP**	**NP**	**SP**	**NP**	**SP**	**NP**	**SP**	**NP**	**SP**	**NP**
Mean	**1.04**	**1.73**	**4.07**	**7.17**	18.6	35.8	2.57	3.62	30.9	59.4	0.821	1.57	10.8	16.1	2.08	6.11	13.2	20.5
Median	**0.980**	**1.62**	**3.86**	**6.70**	17.6	33.5	2.43	3.39	29.2	55.5	0.770	1.47	10.2	15.1	1.97	5.72	12.5	19.2
Min.	**0.631**	**0.850**	**2.48**	**3.52**	11.3	17.6	1.56	1.78	18.8	29.2	0.500	0.77	6.58	7.93	1.26	3.00	8.04	10.1
P75	**1.17**	**1.98**	**4.58**	**8.21**	20.9	41.0	2.89	4.15	34.7	68.0	0.92	1.80	12.1	18.5	2.34	7.00	14.8	23.5
P90	**1.56**	**2.89**	**6.12**	**11.9**	27.9	59.8	3.86	6.05	46.4	99.2	1.23	2.63	16.2	27.0	3.13	10.2	19.8	34.3
P95	**1.68**	**2.96**	**6.59**	**12.2**	30.1	61.4	4.16	6.21	49.9	101	1.32	2.70	17.5	27.7	3.37	10.5	21.4	35.2
Max.	**2.39**	**4.07**	**9.35**	**16.8**	42.7	84.4	5.90	8.54	70.9	140	1.87	3.71	24.8	38.1	4.78	14.4	30.4	48.4
**(B) Deterministic Exposure Analysis at Fixed Rice Consumption and Variable Mycotoxin Concentration.**																		
**Descriptive Statistics**	**AFB_2_**		**AFB_1_**		**NIV**		**DAS**		**FB_1_**		**OTA**		**DON**		**HT2**		**ZEN**	
	**SP**	**NP**	**SP**	**NP**	**SP**	**NP**	**SP**	**NP**	**SP**	**NP**	**SP**	**NP**	**SP**	**NP**	**SP**	**NP**	**SP**	**NP**
Mean	**1.04**	**1.73**	**4.07**	**7.17**	18.6	35.8	2.57	3.62	30.8	59.3	0.821	1.57	10.8	16.2	2.08	6.11	13.2	20.5
Median	<LOD	**1.61**	**4.07**	**5.40**	<LOD	<LOD	<LOD	<LOD	<LOD	<LOD	<LOD	<LOD	<LOD	<LOD	<LOD	<LOD	<LOD	<LOD
Min.	<LOD	<LOD	<LOD	<LOD	<LOD	<LOD	<LOD	<LOD	<LOD	<LOD	<LOD	<LOD	<LOD	<LOD	<LOD	<LOD	<LOD	<LOD
P75	**1.71**	**2.90**	**5.86**	**10.4**	0.00	39.28	<LOD	<LOD	96.6	127	<LOD	<LOD	<LOD	<LOD	<LOD	<LOD	<LOD	<LOD
P90	**2.66**	**4.38**	**10.9**	**16.3**	90.2	133	11.3	14.8	96.6	138	<LOD	<LOD	<LOD	<LOD	<LOD	29.5	78.3	104
P95	**4.39**	**6.69**	**13.3**	**19.0**	124	192	11.3	14.8	104	144	4.07	15.1	124	168	<LOD	58.7	79.5	111
Max.	**5.16**	**7.39**	**14.6**	**42.7**	177	255	20.7	16.4	105	164	**18.3**	**52.4**	128	250	51.5	69.8	190	**250**

PMTDI values (ng/kg b.w. day): AFB_1_ = 0.0001, AFB_2_ = 0.0001, NIV = 700, DAS = 100, FB_1_ = 2000, OTA = 17, DON = 1000, HT2 = 100, ZEN = 250.

## Appendix F

**Table A5:** Deterministic dietary exposures (ng/kg b.w./day) through the consumption of rice contaminated with multiple mycotoxins by children in North and South Punjab of Pakistan, using the lower bound scenario (<LOQ = 0). The calculations are based on (**A**) fixed mean mycotoxin concentration and variable rice consumption in the two regions and (**B**) fixed mean rice consumption and variable mycotoxins concentration in the two regions. Values exceeding the PMTDI (provisional maximum tolerable daily intake) are shown in bold.

**(A) Deterministic Exposure Analysis at Fixed Mycotoxin Concentration and Variable Rice Consumption.**																		
**Descriptive Statistics**	**AFB_2_**		**AFB_1_**		**NIV**		**DAS**		**FB_1_**		**OTA**		**DON**		**HT2**		**ZEN**	
	**SP**	**NP**	**SP**	**NP**	**SP**	**NP**	**SP**	**NP**	**SP**	**NP**	**SP**	**NP**	**SP**	**NP**	**SP**	**NP**	**SP**	**NP**
Mean	**1.05**	**1.79**	**4.12**	**7.43**	18.8	37.1	2.60	3.76	31.2	61.6	0.83	1.63	10.9	16.8	2.11	6.34	13.4	21.3
Median	**0.960**	**1.70**	**3.76**	**7.04**	17.2	35.1	2.37	3.56	28.4	58.3	0.75	1.54	9.98	15.8	1.92	6.00	12.2	20.2
Min.	**0.690**	**0.971**	**2.72**	**4.02**	12.4	20.1	1.72	2.03	20.6	33.3	0.54	0.88	7.23	9.06	1.39	3.43	8.83	11.5
P75	**1.12**	**2.12**	**4.38**	**8.80**	20.0	43.9	2.77	4.45	33.2	72.8	0.88	1.93	11.6	19.8	2.24	7.50	14.2	25.2
P90	**1.34**	**2.54**	**5.26**	**10.5**	24.0	52.7	3.32	5.33	39.8	87.5	1.05	2.32	13.9	23.8	2.69	9.00	17.0	30.3
P95	**1.79**	**2.83**	**7.02**	**11.7**	32.1	58.6	4.42	5.93	53.1	97.2	1.40	2.57	18.6	26.4	3.58	10.0	22.7	33.63
Max.	**2.69**	**3.85**	**10.52**	**15.9**	48.1	79.9	6.64	8.08	79.7	132	2.11	3.51	27.9	36.1	5.37	13.6	34.2	45.8
**(B) Deterministic Exposure Analysis at Fixed Rice Consumption and Variable Mycotoxin Concentration.**																		
**Descriptive Statistics**	**AFB_2_**		**AFB_1_**		**NIV**		**DAS**		**FB_1_**		**OTA**		**DON**		**HT2**		**ZEN**	
	**SP**	**NP**	**SP**	**NP**	**SP**	**NP**	**SP**	**NP**	**SP**	**NP**	**SP**	**NP**	**SP**	**NP**	**SP**	**NP**	**SP**	**NP**
Mean	**1.05**	**1.79**	**4.12**	**7.43**	18.8	37.1	2.60	3.76	31.2	61.6	0.831	1.63	10.9	16.7	2.11	6.34	13.4	21.3
Median	<LOD	**1.67**	**4.11**	**5.60**	<LOD	<LOD	<LOD	<LOD	<LOD	<LOD	<LOD	<LOD	<LOD	<LOD	<LOD	<LOD	<LOD	<LOD
Min.	<LOD	<LOD	<LOD	<LOD	<LOD	<LOD	<LOD	<LOD	<LOD	<LOD	<LOD	<LOD	<LOD	<LOD	<LOD	<LOD	<LOD	<LOD
P75	**1.73**	**3.00**	**5.93**	**10.8**	<LOD	40.7	<LOD	3.45	97.8	131	<LOD	<LOD	<LOD	<LOD	<LOD	<LOD	<LOD	<LOD
P90	**2.70**	**4.54**	**11.1**	**16.9**	91.3	138	11.4	15.4	97.8	143	<LOD	<LOD	<LOD	<LOD	<LOD	30.5	79.3	108
P95	**4.44**	**6.94**	**13.5**	**19.7**	125	199	11.5	15.4	105	149	4.11	15.62	125	174	<LOD	60.8	80.4	116
Max.	**5.22**	**7.66**	**14.8**	**44.4**	179	264	21.0	17.0	106	170	**18.5**	**54.3**	129	260	52.1	72.4	192	**259**

PMTDI values (ng/kg b.w. day): AFB_1_ = 0.0001, AFB_2_ = 0.0001, NIV = 700, DAS = 100, FB_1_ = 2000, OTA = 17, DON = 1000, HT2 = 100, ZEN = 250.
